# Latest Advances in Green Extraction of Polyphenols from Plants, Foods and Food By-Products

**DOI:** 10.3390/molecules30010055

**Published:** 2024-12-27

**Authors:** Andrea Palos-Hernández, Ana M. González-Paramás, Celestino Santos-Buelga

**Affiliations:** 1 Departamento de Química Analítica, Nutrición y Bromatología, Facultad de Farmacia, Campus Miguel de Unamuno s/n, Universidad de Salamanca, 37007 Salamanca, Spain; andreapalos@usal.es (A.P.-H.); paramas@usal.es (A.M.G.-P.); 2 Grupo de Investigación en Polifenoles (GIP-USAL), Facultad de Farmacia, Campus Miguel de Unamuno s/n, Universidad de Salamanca, 37007 Salamanca, Spain

**Keywords:** polyphenols, green extraction, sustainability, bioactives

## Abstract

Phenolic compounds present in plants and foods are receiving increasing attention for their bioactive and sensory properties, accompanied by consumers’ interest in products with health benefits derived from natural rather than artificial sources. This, together with the sustainable development goals for the 21st century, has driven the development of green extraction techniques that allow obtaining these compounds with the safety and quality required to be applied in the food, cosmetic and pharmaceutical industries. Green extraction of natural products involves practices aiming at reducing the environmental impact of the preparation processes, based on using natural or less-polluting solvents, lower energetic requirements and shorter extraction times, while providing greater efficiency in the recovery of target compounds. In this article, the principles of sustainable extraction techniques and the advances produced in recent years regarding green isolation of polyphenols from plants, food and food waste are reviewed.

## 1. Introduction

Phenolic compounds are a large group of plant secondary metabolites, widely distributed in the Plant Kingdom, that comprise several classes of structures, such as simple phenols, phenolic acids, flavonoids, coumarins, lignans and stilbenes. They are produced through the shikimate/phenylpropanoid and the acetate/malonate pathways and can be found in the different parts of the plants (roots, stems, leaves, flowers). In their natural media, they can occur in free forms, but also as glycosylated, acylated or polymerized structures (e.g., tannins or lignin), as well as linked to plant matrix components like carbohydrates or proteins. Phenolic compounds are also abundant in plant foods and derived products, where they contribute to sensory, technological and health properties [1].

The phenolic compounds have recently been used for applications in the food, pharmaceutical and cosmetic industries, owing to their antioxidant, anti-inflammatory and antimicrobial properties, and their association with health-promoting effects in the prevention of highly prevalent chronic diseases, such as cardiovascular and neurodegenerative disorders or type II diabetes [1]. The growing interest in these compounds and their applications is evidenced by the large development of processes for their recovery and isolation from plants, food and food waste.

For years, the extraction has been carried out using conventional extraction techniques, such as maceration, distillation, decoction, heat reflux and Soxhlet extraction, normally using organic solvents, such as ethanol, methanol, ethyl acetate or acetone, alone or in water mixtures. The main drawbacks of these techniques are the use of polluting solvents, energy consumption, long extraction times, toxicity and/or degradation of thermo-sensitive compounds [2]. These disadvantages, coupled with increased environmental awareness and demand for natural and more environmentally friendly products, have promoted the development of new green extraction methods.

Green technologies involve the search for, and development of, more advanced methods that minimize energy consumption and use more ecological solvents, and promote renewable, safer and high-quality natural products [3]. To achieve these goals, the key points are to improve heat and mass transfer rates, reduce extraction times and increase efficiency by reducing instrumentation and labour costs [4].

The aim of this article is to review the main advances and innovations in the development of sustainable extraction techniques for the isolation of phenolic compounds from natural sources and to assess the potential of novel techniques and green solvents to improve the sustainability of extraction processes.

## 2. Sustainable Extraction of Polyphenols

In recent years, increasing environmental awareness has launched research aimed at establishing sustainable extraction methods that produce safe and quality extracts for application in the food, pharmaceutical and cosmetic industries. With these needs in mind, researchers coined the term “green extraction” based on six principles [5]:

- Use of renewable and sustainable bio-resources.
- Use of green solvents or water.
- Lower energy input.
- Coproducts production from waste.
- Minimal number of operation units.
- Obtaining non-denatured and biodegradable extract.

### 2.1. Sustainable Extraction Techniques

Sustainable extraction involves the use of techniques saving time, energy consumption and cost, such as ultrasound-assisted extraction (UAE), microwave-assisted extraction (MAE), pressurized liquid extraction (PLE), and others, as described below.

#### 2.1.1. Microwave-Assisted Extraction

MAE consists of the application of electromagnetic radiation at wavelengths in the microwave range. Microwave radiation is directed at the dipolar molecules by ionic conduction or dipolar rotation, being absorbed and transduced into thermal energy to a varying degree. This leads to stirring of the extraction system (sample and solvent), rapid dielectric heating without thermal gradients and disruption of hydrogen bonds [6]. The improvement in extraction efficiency is due to the fact that the thermal energy causes rupture of the cell membranes, releasing the cell content into the medium [7]. The extraction takes place in three stages: the elevation of pressure and temperature, which causes the separation of the bioactive from the matrix, the distribution of the solvent in the matrix, and the leaching of the solute into the solvent [3]. The dielectric heating of the medium depends on the frequency and power of the microwaves [8]. The use of solvents with a high dielectric constant reduces the heating time, which determines the efficiency of the extraction [9].

The main advantages of using MAE are the reduction of the extraction time, compatibility with natural solvents and efficiency. It also has significant benefits in terms of scalability (safety, cost, energy control and automation) [10]. The main disadvantage is that non-polar solvents cannot be used because of their inability to heat up under microwave radiation [11].

In recent years, several MAE processes have been optimized for the isolation of phenolic compounds from different plant matrices. Interesting results were obtained by Feumba et al. [12], who examined the antioxidant activity and browning behavior of free and bound phenolic fractions of mango, apple, orange and banana peelings subjected to microwave treatment at 720 W for 1, 3 and 5 min. Their results showed that increased microwave blanching was accompanied by a significant increase in the extraction of total phenolics, from both free and bound fractions, and a decrease in browning rates, except for apple peelings.

The phenolic extraction efficiency can be significantly improved by reducing the pH and increasing the power and irradiation time, as demonstrated by Vu et al. [13] for the extraction of phenolic compounds from banana peel. The enhancement in the extraction efficiency of phenolic compounds at increasing extraction times and microwave power was also shown by Adulley et al. [14] on *Persea americana* Mill. seeds. Baltacıoğlu et al. [15] found that MAE caused morphological changes that increased the mass transfer rate, improving the extraction yields of phenolic compounds from tomato. In general, most of the studies consulted on optimizing the extraction of polyphenol-rich extracts by MAE agree that high temperatures of 80–90 °C, moderate powers (about 500 W) and short extraction times (less than 3 min) maximize extraction efficiency [16,17,18,19,20]. As for the solid/liquid ratios, there is a large variability between the values offered by the different authors, with a great influence of the matrix. Examples of polyphenol extraction from different matrices using MAE with conventional solvents are summarized in Table 1. Studies combining microwaves with green solvents are also described in Section 2.3.

#### 2.1.2. Ultrasound-Assisted Extraction

UAE is produced by sound waves with a frequency above the range audible to humans (16–18 kHz), which cause cavitation bubbles that destroy plant cell walls with consequent release of the cellular content [30]. The fundamental mechanism of ultrasound is based on the transformation of electrical energy into mechanical energy through transducers, promoting mechanical vibration at high frequency (>20 kHz). The energy of the waves cannot be directly absorbed by the matrix particles because the wavelengths of ultrasound are much longer than those of biomolecules and macromolecules. The upper frequency limit in liquids and solids is 500 MHz.

The effectiveness of ultrasound lies in the strong shock forces caused by microjets and shock waves produced by ultrasonication of liquids. The isotropic transmission of mechanical energy occurs through rapidly alternating compression/decompression cycles [8]. Rapid high/low pressure oscillations produce vacuum nanobubbles at innumerable nucleation points. Over successive compression–decompression fluctuations, the bubbles accumulate energy until their resistance threshold is exceeded and they violently collapse. Finally, cavitation occurs: the simultaneous implosion of huge numbers of vacuum bubbles produces powerful shear forces that propagate in the form of microjets and shock waves [31]. The power of ultrasonic waves can promote the dissipation of mechanical energy, causing a “heating effect”, consequently increasing the diffusivity in the solid medium [32]. An additional consequence of acoustic cavitation is related to cell and tissue disruption, which may enhance the mass transfer phenomena due to the release of the particles from the plant cell wall, increasing the solute–solvent contact. Moreover, the formation of cavities and microchannels has also been identified as the main reason for the rise in the mass transfer phenomena in food processing assisted by ultrasound [22,33]. UAE is a proven green bio-refining technology, mainly due to its ability to lower cost, reduce operation time, reduce energy consumption and produce higher yields [2,33].

The efficiency of this technique for polyphenol extraction has been shown in different plant matrices such as the edible flower *Clitoria ternatea* [34], foods like *Tetrapleura tetraptera* L. dry fruit [35], turkey berry [36], jackfruit [23] or *Berberis jaeschkeana* fruits [25], medicinal plants like *Empetrum nigrum* [37], and wastes such as spent coffee grounds [38]. In this latter case, the improvement in polyphenol recovery yields increased by 33%, reducing energy consumption by half. In general, it is agreed that modest temperature conditions (40–45 °C), short extraction times (less than 30 min) and moderate power (less than 500 W) are sufficient to increase the polyphenols yield and the antioxidant activity of the extracts [39], although higher power could be used to improve the phenolic extractability and bioaccessibility [13].

The main disadvantage of UAE is that it may cause degradation of specific compounds in the extracts [40]. Thus, while the extraction rate can be improved by the high temperature reached inside the bubbles caused by UAE, such conditions could lead to degradation of phenolic compounds when longer times are used [41]. The effect of ultrasounds on the stability of polyphenols from *Berberis jaeschkeana* C.K. Schneid. fruits was investigated by Belwal et al. [25], showing that rutin, vanillic acid, 3-hydroxybenzoic acid and trans-cinnamic acid were more stable when UAE was applied compared with conventional extraction with methanol, while caffeic, chlorogenic, *p*-coumaric and 4-hydroxybenzoic acids were completely degraded under UAE conditions. The stability of polyphenols after cavitation by UAE depends on the compound, the extraction solvent and the temperature. Studies on young red wines by Celotti et al. [42] showed that increasing ultrasound amplitude resulted in a decrease in the yield of extraction of flavan-3-ols due to chemical degradation, but that it did not affect anthocyanin recapture. Qiao et al. [43] observed that some phenolic acids, like protocatechuic acid or vanillic acid, were stable to UAE, while caffeic and sinapic acids were degraded under ultrasound treatment. Similar observations were made by Belwal et al. [25]. Degradation of (+)-catechin was found by Zhu et al. [44], while Pingret et al. [45] reported that the major polyphenols in apple pomace, i.e., epicatechin or chlorogenic acid, were not degraded. These facts revealed that the degradation does not only depend on the type of phenolic compound, but it is also affected by the extraction conditions. Qiao et al. [43] reported that the intensity of cavitation can be decreased to mitigate its undesired effects on the stability of phenolic acids by increasing the temperature. Similarly, Sun et al. [46] indicated that vapor pressure, which increases with temperature, allows reducing the intensity of cavitation. All in all, the use of ultrasound can improve the extraction yields, but, in many cases, alters the phenolic profile of the extracts obtained.

The UAE conditions used by different authors for polyphenol extraction from distinct matrices studies are summarized in Table 2.

#### 2.1.3. Pressurized Liquid Extraction

PLE, also called accelerated solvent extraction (ASE), is one of the most promising extraction technologies, especially when the extraction is carried out using environmentally friendly solvents. This technique consists of combining high pressures (4–20 MPa) with moderate-to-high temperatures above the normal boiling point of solvents, resulting in the breaking of secondary bonds and thus in the enhancement of the desorption rate and solubilization of matrix-bound species [53]. Extraction can be performed by keeping the solid sample and solvent encapsulated in a container for intervals of a few minutes (static extraction), or in the form of dynamic runs, where the solvent continuously enters the extraction chamber [54]. PLE has proven to be efficient for polyphenol extraction without altering its composition, making it possible to apply the extracts obtained in different health-promoting sectors, such as cosmeceuticals or nutraceuticals. Among its main advantages are the reduction of extraction times, due to the decrease in viscosity and surface tension of the solvents, with a consequent increase in the rate of diffusion, easy automation and good reproducibility, and a decrease in the amount of solvent to be used [55].

On the other hand, the main disadvantages of PLE are the high cost of the required equipment, at both the laboratory and industrial scales, and selectivity, as co-extraction of interfering substances from complex matrices together with the target species is relatively frequent [56].

The efficiency of PLE for polyphenol extraction compared with conventional solvent extraction has been demonstrated by several authors. For instance, Andrade et al. [57] achieved an anthocyanin recovery of up to 88% from *Aronia melanocarpa* pomace using PLE and 1.5% of citric acid as solvent with an extraction time of 45 min. Gómez-López et al. [58] showed that PLE improved the extraction of phenolic bioactives like piscidic acid from fruits of *Opuntia stricta* var. *Dillenii* by 124% using ethanol as a solvent, compared with conventional extraction, also increasing the antioxidant and anti-inflammatory capacity of the extracts. Similarly, Cea et al. [59] obtained extracts from olive pomace using PLE and ethanol as a solvent, three to four times richer in phenolic compounds than the conventional method.

Recent studies using PLE for phenolic extraction from different matrices are summarized in Table 3. In all cases, the solvent used was ethanol, alone or in water mixtures, with extraction conditions strongly dependent on the matrix.

#### 2.1.4. Supercritical Fluid Extraction

Supercritical fluids (SCFs) are substances at a temperature and pressure above their critical point. Under these conditions, there is no distinction between the gas and the liquid phases. The main advantage of SCFs is their versatility, because their density (and thus their solvating power) can be adjusted by changing pressure and/or temperature, which may allow selective extraction of target analytes [67]. Another advantage of supercritical fluid extraction (SFE) is that it permits eliminating or reducing organic solvents, while improving yields using shorter extraction times.

Carbon dioxide is one of the most widely used solvent due to its critical temperature (31.2 °C), which allows a better preservation of thermolabile bioactive compounds compared to other extraction techniques. Furthermore, it is harmless to human health [68], prevents oxidation reactions by avoiding contact with air and it is a readily available and reusable gas [69]. Although supercritical CO_2_ (SC-CO_2_) extraction can be carried out without organic solvents, the addition of a percentage of polar solvents, such as ethanol, can improve the solubility of the compounds, probably due to the formation of hydrogen bridging bonds, which favors the molecular interaction between the co-solvent and the solutes [70].

Several recent studies have evaluated the effects of different types and concentrations of co-solvents of SC-CO_2_ on the extraction yield, composition and antioxidant capacity of phenolic extracts. In general, the use of ethanol as a co-solvent at moderate temperatures (40–60 °C) is the most common approach. Radzali et al. [71] explored the efficiency of ethanol, water, methanol, and ethanol–water and methanol–water mixtures as SC-CO_2_ co-solvents for the extraction of polyphenols from the herb *Labisia pumila*, with 90% ethanol–water (70%) co-solvent providing the highest phenolic yield and antioxidant capacity.

Similarly, Quispe-Fuentes et al. [72] carried out SFE of mandarin peel (var. *Clementina orogrande*) using CO_2_ and CO_2_ with 5% and 10% ethanol, also finding that the use of ethanol improved polyphenol extraction and antioxidant activity of the extracts. Table 4 summarizes the SFE conditions used in recent studies for the extraction of phenolic compounds from different plant sources. In general, most studios operate at moderate temperatures of 50–60 °C, pressures of around 250 bar and organic co-solvent content of less than 20% [71,72,73,74,75,76]. Further improvements of the operating conditions might focus on the reduction of the amount of organic co-solvent and the optimization of pressure and temperature conditions.

#### 2.1.5. High-Voltage Electrical Discharges (HVED)

Extraction assisted by high-voltage electrical discharges (HVED) is a non-thermal technique, based on the phenomenon of electrical breakdown of a liquid, which improves the mass transfer of natural ingredients in liquid at room temperature [82]. This extraction technique involves the application of pulsed fast discharge voltages (typically 20 to 80 kV/cm electric field strength) across an electrode gap below the surface of suspensions, generating the acceleration of electrons, which obtain adequate energy to excite the water molecules [83]. The process consists of two stages: streamer discharge (pre-breakdown phase) and arc (breakdown phase) discharge processes. In both stages, shock waves, bubbles, UV radiation and radicals are generated, these effects being much more intense in the breakdown phase [84]. The extraction of bioactive compounds occurs due to mechanical damage and disintegration of the cell walls caused by shock waves, cavitation and UV light [83,85]. The main advantages of HVED compared to conventional solvent extraction are shorter processing times, higher yields, lower energy consumption, fewer impurities in the extract and less damage to the activity and structure of the biologically active components [86].

The efficacy of this technique for phenolic extraction has been shown in several plant matrices, always obtaining extraction yields significantly higher compared to conventional methods. Nutrizio et al. [87], in the extraction of oregano samples with 50% ethanol, achieved yields of phenolics between 2.19% and 34.04% higher than infusion, and between 6% and 91% higher than maceration, depending on the voltage and treatment time used for HVED. For their part, Hejazi et al. [19] showed that an increase in energy intake led to a pronounced increase in the extraction of total phenolics from red onion peel.

Table 5 collects the conditions used by different authors for HVED extraction of phenolic compounds from plant sources. Most studies achieve good extraction yields by working with voltages of 20–25 kV in short periods of time (less than 10 min). The critical points of this extraction process, according to the studies, are voltage, time and organic solvent content.

#### 2.1.6. Enzyme-Assisted Extraction (EAE)

Phenolic compounds are often retained in cell walls by hydrogen and hydrophobic bonds within the polysaccharide–lignin network of the wall structure [96]. Pre-treatment with a degrading enzyme or different combinations of polysaccharide-degrading enzymes disrupts the cell envelope and releases the network of wall-bound compounds, ameliorating the permeability and extractability (solvation and mass transfer) of phenolic compounds that are non-extractable with conventional extraction [97]. Enzymes used in this process include cellulases, hemicellulases, xylanases, proteases, α-amylases, β-glucosidases and pectinases [98]. The efficiency of extraction depends on the solvent system, temperature, type of enzyme activity, enzyme loading, time of extraction, substrate availability and pH conditions [97]. pH and temperature are particularly critical for activating the catalytic potential. Extractions are usually performed at low pH, as acidic media favor the cleavage of the secondary bonds linking phenolics to cell wall components. Moreover, as particle diameter decreases, the accessibility of enzymes to susceptible bonds increases, and the diffusion pathways to the solvent of the released species are reduced [8].

The benefits of enzyme-assisted extraction include shorter extraction time, reduced solvent needs, improved quality of extracted compounds, gentler conditions, and less waste [98,99]. Among the possible disadvantages are the fact that some enzymes can alter the phenolic profile and the negative interference of improper enzyme activities from the plant matrix [100]. Recent studies using EAE for phenolic extraction are collected in Table 6.

#### 2.1.7. Combined Novel Methods

The use of combined extraction methods is becoming an alternative to reduce costs and maximize yields. The positive effect of the combination of two extraction methods has been tested by Pereira et al. [106], who used supercritical extraction with pressurized liquids to recover bioactive compounds from *Butia capitata*, achieving a 1.4-fold increase in performance. For their part, Pereira et al. [107] integrated ultrasound-assisted pressurized liquid extraction (UAPLE) and nanofiltration in 70% ethanol, obtaining promising results in the isolation and concentration of phenolic compounds from passion fruit peel.

However, Cheng et al. [23] observed no positive effect on the concentration of phenolic compounds in extracts obtained from jackfruit combining MAE and UAE. However, there is not enough data to clearly establish the advantages and disadvantages of combining different extraction methods.

Combinations of different extraction methods and green solvents are discussed in Section 2.3.

#### 2.1.8. Implications for the Selection of the Best Extraction Method

Different methods of sustainable extraction are currently available, as discussed throughout this section. Their efficacy compared to conventional methods has been widely demonstrated. As shown in Table 7, all have advantages and limitations, but the approaches to sustainability have in common the reduction of extraction times and the use of moderate operating conditions, to reduce energy consumption and operating costs.

However, the choice of the best extraction method is not simple and has to consider the nature of the compounds and the matrix. Kaderides et al. [26] compared the efficiency of UAE and MAE on the extraction of polyphenols from pomegranate peels, obtaining a 1.7-times higher yield in half the time using MAE; the difference was attributed to the intense cell destruction of MAE-treated plant material, as checked by scanning electron analysis. Cheng et al. [23] and de Jesus et al. [110] also found higher recovery of phenolic compounds from jackfruit pulp and leaves of the medicinal plant *Lantana camara*, respectively, using MAE rather than UAE. In this latter case, the extraction yield of polyphenols obtained by MAE increased by 6% and the processing time was reduced from 25 to 15 min, compared to UAE, but using a higher percentage of ethanol (53% vs. 35%). Increased solvent and liquid-to-solid ratio requirements of MAE in relation to UAE were also reported by Irakli et al. [111] when optimizing the parameters for phenolic extraction from sage residues obtained after essential oil distillation. Therefore, to select one or another technique, not only the extraction yields but also the operating conditions must be considered, since, for example, a greater solvent requirement would make the process less sustainable. On the other hand, UAE could affect the phenolic profile, although by controlling the temperature, satisfactory yields can be obtained at reduced times without altering the phenolic composition, as demonstrated Aliaño et al. [102] and Vidana et al. [21] on the extraction of anthocyanins from blackcurrant and blue pea flower, respectively. On the other hand, Vidana et al. [21] found MAE and UAE more effective than enzyme-assisted extraction in recovering anthocyanins from blue pea flower [21], but not in the case of black goji berry [22], where pectinase-assisted extraction increased yields by up to 10 compared to MAE and UAE. These contrasting results reinforced the importance of the plant matrix in the selection of the extraction method.

PLE is another technique that usually offers favorable results in comparative studies, although the high equipment costs can be a major disadvantage. For instance, Frohlich et al. [60] observed that the extraction yield was almost doubled in the recovery of antioxidants and eugenol from leaves using PLE than SFE. Similar observations were made by Santos et al. [62], who obtained better performance in the extraction of total phenolics and antioxidant activities from feijoa leaves by PLE compared to SFE. Some recent works comparing extraction techniques are shown in Table 8.

### 2.2. Green Solvents

The previous sections have been mostly focused on extraction procedures using conventional organic solvents, usually methanol or ethanol.

The environmental impact, together with the toxicity of some of these solvents, has promoted the search for other, more environmentally friendly, solvents that allow the safe application of extracts in the food, pharmaceutical or cosmetic industries. Next, the use of solvents of natural origin, that are non-toxic and that can provide benefits in the extraction of target compounds and/or particular characteristics to the extracts obtained (e.g., antioxidant, antibacterial or anti-inflammatory properties) is discussed. Among them, the most promising ones are bio-based solvents (BBSs), natural deep eutectic solvents (NADES), non-ionic surfactant mixtures and ionic liquids (ILS).

#### 2.2.1. Bio-Based Solvents

The term bio-based solvents (BBSs) includes many biomolecules derived from renewable sources that are benign and less toxic than the usually employed volatile organic solvents, thus being an option for the extraction of bioactive compounds from natural matrices [115]. They can be sorted into three categories depending on the agro sector where they are produced: cereal/sugar, oleo-proteaginous and wood [67].

Due to their heterogeneity, they have no common properties, and the selection of the most suitable one must be made according to the type of compounds to be extracted, although, in general, extraction is favored by the presence of a lower number of hydroxyl groups, higher polarity and simpler structures that minimize steric hindrance [116].

Despite their potential, BBSs have hardly been investigated for the extraction of phenolic compounds. Vieira et al. [117] tested a series of alkanediols (1,2-ethanediol, 1,2-propanediol,1,3-propanediol, 1,3-butanediol, 1,2-pentanediol, 1,5-pentanediol and 1,2-hexanediol) for the extraction of polyphenols from leaves of *Juglans regia* L., a rich source of this class of bioactives. The solvents that showed the best combined results were 1,2- and 1,3-propanediol, with results close to those obtained with ethanol. Ozturk et al. [118] compared the performance of a variety of BBSs, i.e., cyclopentylmethyl ether (CPME), ethyl lactate (EL), isopropyl alcohol (IPA), polyethylene glycol 300 (PEG 300), isopropyl acetate (IAc), dimethyl carbonate (DMC), methyl ethyl ketone (MEK), 2-methyltetrahydrofuran (2-MeTHF) and ethyl acetate (EAc) for the sustainable valorization of orange peel waste through limonene extraction. The best results were obtained using CPME, with an increase in the extraction yields up to 80% compared to hexane. Cravotto et al. [119], in olive pomace, demonstrated that the use of dry 2-methyloxolane (2-MeOx) was more efficient than hexane in terms of extraction yields, increasing the total recovery of phenolic compounds from 0.86 to 22 mg GAE/g. Although oleacein and oleocanthal were the two main phenolic compounds, in terms of concentration, the extracts were characterized by the significant presence of other phenolics like tyrosol, hydroxytyrosol, flavonoids (e.g., luteolin, apigenin), lignans (e.g., pinoresinol, acetoxypinoresinol) and phenolic acids (e.g., caffeic and p-coumaric acids).

#### 2.2.2. Natural Deep Eutectic Solvents (NADES)

NADES are liquids consisting of a hydrogen-bond acceptor (HBA), such as a non-toxic quaternary ammonium salt (e.g., choline chloride), and an uncharged hydrogen-bond donor (HBD), usually amines, sugars, alcohols and carboxylic acids [120]. This type of solvent has generated great interest due to their versatile physicochemical properties, acceptable costs, easy preparation, reduced toxicity and environmental impact and less energy consumption. However, their high viscosity and density, and very low vapor pressure, pose some obvious limitations [121]. Indeed, water content, viscosity, polarity and density are key physicochemical characteristics that influence the extraction efficiency of NADES. Density can be decreased by a moderate increase in water content, which can be beneficial for extraction processes, although a significant increase in water content can break the bonding network of the NADES, impairing extraction [122,123]. Viscosity can be decreased by increasing temperature, which improves the extraction efficiency; however, above certain values, temperature leads to degradation or isomerization of phenolic compounds [122,123,124]. The polarity depends on the components that constitute the NADES. Since an increase in temperature tends to decrease the polarity, by adjusting temperature, it could be possible to align the polarity of the solvent with that of the solute, increasing solubility and improving extraction efficiency [125].

The improvement in extraction yields has been demonstrated by several authors. In the experiments of Santos-Martín et al. [126] on blueberry leaves, different combinations of NADES, such as lactic acid, sodium acetate, and water (3:1:2) or choline and oxalic chloride (1:1), obtained richer polyphenol extracts of 142.5 and 195.5 mg GAE/g dried mass, compared to 86.9 mg GAE/g dried mass obtained using methanol (80%). Similar results were obtained by Duarte et al. [127], who managed to triple the recovery of phenolics from the by-product of Purple Araçá (*Psidium myrtoides*) using choline chloride and glycerol (1:1). Another example of improved extraction yields by the use of NADES are the results of Koraqi et al. [128] on *Rosa damascena* Mill., in which the solvent consisting of lactic acid, citric acid, and glycerol (3:1:2) recovered 673.1 mg GAE/g compared to 203.3 mg GAE/g obtained using methanol.

Interestingly, an improvement in the bioactivity of NADES-based extracts was reported by some authors. Santos-Martín et al. [126] observed that the antioxidant activity extracts obtained with lactate, sodium acetate and water (3:1:2) was almost three times higher than those of methanolic-extracts after the optimization of the process. Similarly, Mansinhos et al. [129] found higher polyphenol contents and antioxidant capacity, as assessed by DPPH and ABTS assays, in extracts of *Lavandula pedunculata* subsp. *Lusitanica* prepared with choline chloride and urea (1:2) than in those obtained with conventional organic solvents. However, no enhancement of antioxidant activity, but increased tyrosinase and diabetic enzyme inhibitory properties, were described by Zengin et al. [130] for NADES-based extracts obtained from *Cytinus hypocistis* compared to those obtained with traditional organic solvents. On the other hand, Jurić et al. [131] observed that *Mentha piperita* extracts based on choline chloride and organic acids such as citric and malic acids showed increased antimicrobial activity, inhibiting the growth of bacteria such as *Pseudomonas aeruginosa*, *Staphylococcus aureus*, *Escherichia coli* or *Salmonella enterica* at lower concentrations than ethanol-based extracts.

The processes of optimization with NADES are aimed at selecting the appropriate solvent composition (HBA and HBD) according to the type of phenolic compound. For instance, it has been reported that the extraction of anthocyanins is favored by the combination of choline chloride with an organic acid, such as malic, lactic or oxalic acids, while the extraction of flavanols benefits from the combination of choline chloride and some alcohols, such as propylene glycol [121]. Research has also been focused on evaluating the stability of the extracts obtained. As an example, greater extraction yield and compound stability was obtained by Gómez-Urios et al. [132] using choline chloride–malic acid (ChCl:MalA) for the extraction of polyphenols from orange peel compared with sugar or polyalcohol-based NADES. The extraction efficiency can be improved when NADES are combined with a supporting extraction technique such as MAE or UAE (Section 2.3). Evidence about the use of natural deep eutectic solvents for the extraction of polyphenols from natural sources can be found in a recent review by Palos-Hernández et al. [121], and further information about the combination of NADES with other extraction techniques is described in Section 2.3 of this review.

#### 2.2.3. Non-Ionic Surfactant Mixtures

Surface-active agents, or surfactants, are amphiphilic molecules consisting of a polar “head” connected to a hydrophobic “tail” group. They possess different structures (e.g., polysorbates, ethoxylate alcohols, polyethylene glycols) that are commercialized under different denominations (Tween 20, Triton X-100, Span 20, etc.). In polar media, these molecules are grouped together with the hydrophobic part oriented towards the center, with lower porosity for water molecules [133]. They can provide higher recovery rates of bioactive phytochemicals compared to conventional solvents [134], as they allow for dissolving poorly water-soluble substances due to the formation of micelles, with the polarity and chemical structure of the substances determining their location in the micelle [135]. After extraction, the surfactants remain in the final extracts as part of their integral composition [136], which avoids further purification processes and solvent evaporation, thus reducing energy expenditure.

Although some types of surfactants might be harmful to health and the environment [137], most non-ionic aqueous surfactant solutions are considered relatively non-toxic or harmless solvents with adequate stability and compatibility [137], representing a cost-effective, user-friendly, biocompatible and environmentally sustainable alternative to organic solvents for the extraction of polyphenols [137,138,139]. A disadvantage of this type of solvent is the formation of turbidity once a critical micellar concentration is reached, making photometric analysis of compounds like anthocyanins impossible [140].

Non-ionic surfactants appear as an interesting alternative for the extraction of phenolic compounds, although not many studies have been carried out so far. The choice of an effective surfactant for micelle-mediated extraction should consider the nature of the matrix and the phenolic compounds to be extracted. Sazdanić et al. [136] showed that the chemical structure of the surfactant influences the mechanism of solubilization of polyphenols and that the rate of extraction also depends on the concentration and the solvent/material ratio. Skrypnik et al. [137] evaluated the extraction yields of phenolic compounds from apple pomace using five non-ionic solvents (Tween 80, Triton X-100, Span 20, Tween 20, 70% ethanol) and water, Tween 80 being the one with the best extraction efficiency, reaching 98.73% of the predicted total phenolic value. The use of Tween 80 was also proposed for the extraction of antioxidant polyphenols from rattan tea (*Ampelopsis grossedentata*) [141], while Brij-58 and Brij-35 solutions were found to be the most effective in the extraction of phenolic compounds from fruits [136,137,138,139,140,141,142], and Triton X-100 for the extraction of conjugated phenolic compounds from black tea [143]. The potential of aqueous solutions of different mixtures of Brij S20 (BS20) and poloxamer 407 (P407) for the extraction of polyphenols from red grape pomace was examined by Atanacković et al. [139], who found that the best results were obtained for a combination of BS20/P407 (1:1), with recoveries up to 54.7 mg GAE/g dw. Mohd Maidin et al. [144] investigated the stability of anthocyanins obtained from grape pomace by hydroalcoholic extraction with a surfactant-based separation (Tween 20), observing that anthocyanin stability increased at higher Tween 20 concentrations. The non-ionic surfactant PEG 8000 was used by Yu et al. [145] for the extraction of bioactive compounds from fig tree (*Ficus carica* L.) leaves at a pilot scale, reaching extraction yields of caffeoylmalic acid, psoralic acid-glucoside, rutin, psoralen and bergaptene of 9.7 mg/g, 5.9 mg/g, 4.8 mg/g, 15.7 mg/g and 3.5 mg/g, respectively.

#### 2.2.4. Ionic Liquids

Ionic liquids (ILs) are salts whose melting point or glass transition temperatures are below 100 °C, and which occur in a liquid state at room temperature [146]. They are generally composed of organic cations (imidazolium, pyridinium, phosphonium, pyrrolidinium, piperidinium, morpholinium or colinium) and a wide range of organic or inorganic anions [147]. The advantages offered by these solvents are provided by their physicochemical properties: low vapor pressure, thermal stability, non-flammability, a wide electrochemical window and tunable miscibility. These characteristics depend on the combined cationic and anionic components. Some physicochemical properties (thermal stability and miscibility) are mainly affected by the anion present, while other properties (viscosity, surface tension and density) vary depending on the length, symmetry and/or shape of the cationic alkyl chain used [148].

ILs have not been extensively tested for the extraction of phenolic compounds from plant matrices. The approaches carried out are in combination with other sustainable extraction techniques as reviewed in Section 2.3.

Further information on the applications of IL, DES, and NADES for the extraction of bioactive compounds from natural sources can be found in the comprehensive review published by Benvenuti et al. [149].

### 2.3. A Step Closer to Sustainability: Combination of Novel Techniques and Green Solvents

A further step in the development of effectively sustainable extraction methods consists of combining novel techniques that minimize costs and energy consumption with the use of green solvents that reduce pollution. Combinations of UAE, MAE and PLE with NADES are among the most employed ones (Table 9). For instance, the extraction of phenolic compounds from date seeds using seven NADES coupled with UAE was explored by Airouyuwa et al. [150]; all NADES offered better results than conventional solvents, with choline chloride–lactic acid (ChCl:LA) showing the best efficiency for the recovery of phenolic compounds and the highest antioxidant activity. Similarly, Popovic et al. [151] investigated several NADES based on choline chloride for the extraction of polyphenols, and especially anthocyanins, from sour cherry pomace in combination with MAE. All the NADES systems checked were more efficient for anthocyanin extraction than the conventional solvent (0.1% HCl in methanol), with ChCl:MalA having the best performance, and MAE allowing to reduce the extraction time to less than 5 min. For their part, Benvenutti et al. [152] recovered anthocyanin-rich fractions from jaboticaba peels by combining aqueous solutions of deep eutectic solvents and PLE, obtaining anthocyanin yields up to 50% higher than with conventional solvents; newly, ChCl:MalA led to the best performance and the highest anthocyanin stability. Only one study has been found combining HVED with NADES [153] to improve the extraction kinetics and polyphenol yields from grapefruit peels, concluding that the energy expenditure was reduced when the subsequent solid–liquid extraction was performed in 20% (*w*/*v*) aqueous glycerol or NADES (lactic acid–glucose) instead of water.

There are also approaches combining ILs, BBSs or non-ionic surfactants with different extraction techniques. Silva et al. [154] conducted a comprehensive study to valorize waste from the kiwifruit industry. Firstly, they selected a BBS optimizing the extraction according to its composition (gamma-valerolactone/ethanol in a ratio of 7:3, *w*/*w*), and then they combined it with UAE and MAE, the MAE-BBS combination being the most promising one in terms of extraction efficiency (29.7 mg GAE/g dw) and bioactive properties. Ferreira et al. [155] combined ILs (1-butyl-3-methylimidazolium tetrafluoroborate, [BMIM][BF4], and 1-butyl-3-methylimidazolium chloride, [BMIM][Cl], with UAE to extract antioxidant phenolics from guava co-products, obtaining high recoveries of condensed tannins and ortho-diphenols. A non-ionic surfactant (PEG 8000), together with MAE, was successfully employed by Yu et al. [145] to extract bioactive compounds (polyphenols and furanocoumarins) from fig (*Ficus carica* L.) leaves.

A few authors have also combined different extraction techniques. Thus, Lou et al. [156] assayed the combination of ultrasounds and microwaves (UMAE) with ionic liquids (1-alkyl-3-methylimidazolium derivatives) for the extraction of phenolic compounds from burdock leaves (*Arctium lappa* L.), achieving an improvement in the extraction efficiency of up to 17% and a reduction in extraction time from 5 h to 30 s, compared with conventional heat–reflux extraction. Andrade et al. [57] drew on PLE assisted by ultrasound to isolate anthocyanins from black chokeberry (*Aronia melanocarpa*) pomace using 1.5% citric acid as solvent, obtaining a recovery of about 88% of the theoretical content of total anthocyanins. The combination of ultrasounds and microwaves with several NADES was assayed by Vo et al. [157] for the extraction of polyphenols and terpenoids from *Abelmoschus sagittifolius* roots, finding that citric acid/glucose and lactic acid/glucose, at a molar ratio of 2:1, were the most suitable solvents for the efficient recovery of polyphenols and terpenoids, respectively.

A summary of methods combining extraction techniques and green solvents for the recovery of phenolic compounds is included in Table 9.

**Table 9:** Combined techniques used for green extraction of phenolic compounds.

Matrix	Solvent/Enzyme	Extraction Conditions	TPC (mg GAE/g Dry Mass)	Reference
**MAE-SRF**				
Fig (*Ficus carica* L.) leaves	PEG 8000	39.53 °C, 10.25 min, 19.95 mL/g	39.6	[145]
Discarded red beetroot	PEG 4000	160 °C, 5.3 min, 8.4 g/L	1789 *	[158]
**MAE-ILs**				
*Nelumbo nucifera* Gaertn.	[C4MIM][BF4] 1.5 M	10 mL/g, 90 s	-	[159]
	[C6MIM][BF4] 1 M	15 mL/g, 90 s	-	
**MAE-NADES**				
Cherry pomace	ChCl:MalA	180 W, 30 s	-	[151]
Hazelnut (*Corylus avellana* L.) pomace Grape waste *Eugenia uniflora* L.	Choline chloride:1,2-propylene glycol	4% water, 18 mL/0.1 g, 92 °C, 38 min	-	[160]
	Betaine:1,2-butanediol (1:4)	3 min, 100 °C	43.7	[161]
	ChCl:lactic acid 1:3	20% water, 39 °C, 5 min, 800 W	-	[162]
**MAE-ENZ**				
Grape (*Vitis vinifera*) seed	Ethanol, ammonium sulfate, pectinase	2.5% ethanol/20% ammonium sulfate, pectinase 540 U/g, enzymatic pH 4.5, 180 W	125	[163]
Olive pomace	Water	15 mL/g, 60 °C, 17 min, 60 °C, 5 min ramp time	272	[164]
**UAE-SRF**				
Fig (*Ficus carica* L.) leaves	PEG 8000	40 °C, 10 min, 250 W	32.0	[145]
Cili leaves (*Rosa roxburghii* Tratt)	Tris (2-hydroxyethyl) ammonium palmitate (THAP)	20 mg/mL, 120 min, 54 mmol/L CRS	-	[165]
Pomegranate peel	Dimethyl sulfoxide (DMSO)	0.6% DMSO, 50 °C, 150 rpm, 90 min	43.6 **	[134]
**UAE-ENZ**				
Dry biomass of *Arthrospira platensis*	Lysozyme	0.6% enzyme, 16 h incubation	92.7	[166]
*Gymnema sylvestre*	Enzyme cocktail	150 min, 64.80 °C, pH 5.64, 7.49 mL enzyme	109	[167]
**UAE–NADES**				
Palm (*Phoenix dactylifera* L.) seeds	Choline chloride/lactic acid	70% NADES, 0.03 g/mL, 5 min, amplitude of 90%	146	[168]
Mangosteen (*Garcinia mangostana* L.)	Lactic acid-1,2/Propanediol	0.15 g/mL, 30.3% water, 57.5 °C, 9.1 min		[169]
*Lavandula angustifolia* flowers	Choline chloride/glycerol	Amplitude of 60%, 60 °C, 17.5 min, 0.03 g/mL, 33.5% water	50.5	[170]
Apple pomace	Choline chloride/glycerol (1:2)	40 min, water 30%, solid/liquid ratio 1:30, 40 °C, acoustic intensity 83.2 W/cm^2^ and duty cycle 75%	5.6	[168]
	Choline chloride/lactic acid (1:3)		5.1	
Fruit of *Melia azedarach*	Glycerol-choline chloride	50% NADES, 46.4 °C, ultrasound amplitude (100%, 130 W power)	9.2	[171]
Inflorescences of *Helichrysum arenarium* L.	Choline chloride-lactic acid (1:4)	85 min, 38% water	-	[124]
Mango peels	Lactic acid/glucose (5:1)	20% water, 50% duty cycle, 2 W/cm^3^ acoustic density, 30:1 (*v*/*w*) liquid/solid ratio, 0.3 mm particle size and 30 min	69.9	[172]
Cherry pomace	ChCl:MalA	180 W, 30 s	-	[151]
Apple pomace	Choline chloride/glycerol (1:2)	30% water, 40 min, 30 mL/g, 75% duty cycle, 40 °C, acoustic intensity 83.2 W/cm^2^	5.6	[168]
**UAE-IL**				
Chestnut shell	1-Butyl-3-methylimidazolium acetate [BMIM]OAc	36.26 mL/g, 80 °C, 350 W, 50 min	71.6	[173]
Guava (*Psidium guajava* L.)	1-Butyl-3-methylimidazolium tetrafluoroborate [BMIM][BF4]	0.02 g/mL, 25 min, 45 °C, 2.5 mol/of [BMIM][BF4]	1.2	[157]
**PLE-NADES**				
Brazilian berry processing by-product	ChCl:Propylene Glycol (ChCl:Pro)	ChCl:Pro 1:2, 47% NADES, 3 mL/min, 90 °C, 5100 bar, 12 min	85.7	[174]
**SBC-IL**				
Brown seaweeds *Saccharina japonica*	1-Butyl-3-methylimidazolium tetrafluoroborate [BMIM][BF4]	175 °C	-	[175]
**UMAE-IL**				
Burdock leaves	1-butyl-3-methylimidazo-lium bromide [BMIM]Br	30 s, 400 W microwave power, 50 W ultrasound power	-	[156]
**UMAE-NADES**				
*Abelmoschus sagittifolius* (Kurz) Merr Roots	Citric acid/glucose (1:2)	40 mL/g L, 5 min sonication, 1 min	27.1	[157]
**EAE-SFE**				
Pomegranate peel	SC-CO_2_-ethanol and enzyme cocktail (cellulase, pectinase, and protease; 50:25:25)	3.8% enzyme cocktail, 49 °C, 85 min, pH 6.7	301	[93]
**HVED-NADES**				
Grapefruit peel	Lactic acid/glucose	10 mL/g, 40 kV, 0.5 Hz, distance electrodes of 9 mm	-	[153]
**UAE-PLE**				
*Aronia melanocarpa* pomace	1.5% Citric acid	70 °C, 1.5% citric acid 180 bar, 200 W, 45 min	-	[57]

* mg GAE/g fresh mass; ** mg GAE/L.

### 2.4. Final Considerations on the Selection of a Sustainable Extraction Method

There is no procedure that can be recommended for the extraction of phenolic compounds in a general way. The choice will depend on the nature of the matrix and the type of compounds to recover, as well as the intended use of the extracts, which will determine the solvent and/or the extraction technique. From the literature review, it appears that MAE and PLE combined with novel green solvents could be suitable alternatives for efficient polyphenol extraction, NADES being the most explored solvents, while comparative studies combining BBSs, surfactants and ILs have hardly been found. Several examples are given below.

Ming-Zhu et al. [40] found that a MAE–NADES combination was more suitable than UAE–NADES for the extraction of mulberry polyphenols. They attributed it to the fact that microwave radiation can penetrate the plant material and heat it from both the inside and outside, triggering cellular breakdown and shortening the dissolution time of the target components. Abdelrahman et al. [176] found that MAE–NADES extracts from mild date palms were richer in phenolic compounds and possessed higher antioxidant activity than those obtained by UAE–NADES. Similar observations were made by Fan et al. [177] using ILs for the recovery of verbascoside from *Rehmannia* roots, and by Silva et al. [154] using BBSs for the extraction of phenolic compounds from kiwifruit waste.

Grisales-Mejía et al. [178] compared the efficiency of PLE and UAE in the extraction of polyphenols from Hass avocado residues, finding that PLE achieved better results when combined with choline chloride-fructose or lactic acid as solvents. The difference in extraction efficiency was explained as high pressures allowing working close to the boiling point of the solvents, reducing viscosity and surface tension and weakening the interactions between the analyte and the matrix. Vo et al. [179] went a step further and compared the efficiency of NADES combined with UAE, EAE, ultrasonic–enzymatic-assisted extraction (UEAE), enzymatic–ultrasonic-assisted extraction (EUAE) and simultaneous ultrasonic–enzymatic-assisted extraction (SUEAE) in the recovery of flavonoids from tea leaves. They concluded that UEAE was the most efficient extraction approach, probably because after treatment with cellulase, the particle size decreased, reducing the cavitation effect when ultrasound was applied. This effect was not observed in SUEAE and EUAE due to the denaturation of the enzyme, which reduces cellulose hydrolysis, leading to less degradation of the cell wall.

All in all, the selection of the most appropriate procedure for the efficient green extraction of phenolic compounds from natural sources remains a challenge, so that comparison of different approaches on the same matrix should be checked to decide whether to opt for one method or another.

### 2.5. Scalability

One of the most important challenges to advances in this field is the analysis of the scalability of sustainable extraction processes. Although MAE and PLE are considered scalable techniques to implement in a pilot plant or on an industrial scale, there are not many studies that explore the subject in depth. In the case of MAE, some scalability studies have been published using green solvents. Panić et al. [180] extracted polyphenols from grape pomace on a larger scale by combining ultrasonic pretreatment and subsequent microwave extraction using choline chloride and citric acid as solvent. The results obtained were similar to those obtained in the laboratory, demonstrating that this technique is promising for industrial scale. Similar observations were made by Cui et al. [181], who, after optimizing the MAE–NADES extraction process for polyphenols in buckthorn leaves, carried out a pilot plant trial using 500 g of leaves, obtaining the predicted results. A few more examples of pilot plant tests applying MAE–NADES can be found in the review made by Tapia-Quirós et al. [182]. No studies have been found on the scalability of PLE combined with green solvents. Viganó et al. [183] analyzed the scalability of the passion fruit bagasse extraction process using conventional organic solvents combined with a sequential multistage (SFE + PLE) or single-stage PLE process, using 1, 5, 50 and 500 L extraction vessels, obtaining that the increase in scale led to a decrease in manufacturing cost. Indeed, more studies are needed to analyze the factors involved in the scalability of sustainable extraction processes.

## 3. Conclusions

The health benefits of phenolic compounds, together with the demand for natural products and greater environmental awareness, are leading to increased research in sustainable extraction technologies. The search for, and development of, sustainable extraction methods for the isolation of phenolic compounds from plants, food and food waste involves using non-toxic solvents, ensuring high yields, reducing the amount of solvent to be used and reducing the time and energy consumed in the process. The application of green extraction techniques should not only take into account environmental concerns but also contribute to preserving the bioactivity and nutritional value of the extracted phenolic compounds.

Green solvents, such as water, supercritical carbon dioxide (SC-CO_2_) or natural deep eutectic solvents (NADES) have emerged as promising candidates for extracting phenolic compounds due to their environmentally benign nature and the ability to be obtained from renewable resources. So far, there is a large number of studies focused on this objective, and their results indicate that methods combining novel extraction techniques and green solvents may be a promising alternative to conventional extraction methods based on organic solvents. However, practically none of them evaluate the applicability of these techniques on an industrial scale. Moreover, standardization of the extraction technique may be complicated by the variability in phenolic composition of different matrices and its consequent implications on extractability. Therefore, it will be necessary to continue exploring further combinations of the different extraction techniques and green solvents, as well as to evaluate their effective applicability to obtain polyphenol-rich extracts that can be applied in the food, cosmetic or pharmaceutical industries.

## Figures and Tables

**Table 1:** Recent applications of MAE to polyphenol extraction.

Matrix	Solvent	Extraction Conditions	TPC (mg GAE/g Dry Mass)	Reference
**Plants**				
Blue pea flower *Clitoria ternatea* cv.	Water	15 mL/g, 50 °C, 30 min, 400 W	110.4	[21]
Ground ivy (*Glechoma hederacea* L.)	Water	100 mL/g, 90 °C, 4.93 min	48.6	[16]
Nerium oleander leaves	Ethanol (75%)	20 mL/g, 500 W, 1 min	25.8	[20]
Carica papaya leaves	Ethanol	12 mL/g, 50 °C, 3 min, 420 W	102.6	[17]
Fenugreek seeds	Ethanol (≈64%)	0.09 g/mL, 2.84 min, 572.50 W	81.9	[18]
**Food**				
Black goji berry (*Lycium ruthenicum*)	Hot water	15 mL/g, 50 °C, 30 min	69.7	[22]
Jackfruit (*Artocarpus heterophyllus* Lam.) pulp	Ethanol (60%)	0.03 g/mL, 165 s, 550 W	2.4	[23]
*Robinia pseudoacacia* wood	Water	230 °C, 0.25 min	80.3	[24]
Tomato	Methanol (80%) HCl (1%)	90 s, 900 W	436 *	[15]
Red onion	Ethanol (70%)	25 mL/g, 700 W, 65 s	10.9	[19]
Fruits of *Berberis jaeschkeana*	Methanol (80%) HCl 0.1 N	40 mL/g, 670 W, 5 min	108.9	[25]
**Food Waste**				
Pomegranate peel	Ethanol (50%)	60 mL/g, 600 W	-	[26]
Sunflower by-product	Ethanol (70%)	10 mL/g, 30 s, 100 W	12.9	[27]
Banana peel	Water	50 mL/g, pH of 1, 6 min, 960 W	50.6	[13]
Chestnut waste	Water	50 mL/g, 107 °C, 5 min	344	[28]
Lime peel waste	Ethanol (55%)	45 s, 140 W, 8 repeats	59.3	[29]

* mg GAE/kg fresh weight.

**Table 2:** Recent applications of UAE to polyphenol extraction.

Matrix	Solvent	Extraction Conditions	TPC (mg GAE/g Dry Mass)	Reference
**Plants**				
*Ilex paraguariensis* (St. Hil.) leaves	Ethanol (50%)	10 mL/g	0.15	[47]
*Croton heliotropiifolius* Kunth leaves	Ethanol (≈38%)	11.4 mL/g, 54.8 °C, 39.5 min	-	[48]
Leaves of *Lobelia nicotianifolia*	Methanol (≈75%)	62.72 °C, 9.44 min	23.8	[49]
*Clitoria ternatea*	Ethanol (60%)	45 °C, 30 min, 350 W	27.4	[34]
**Food**				
Jackfruit (*Artocarpus heterophyllus* Lam.) pulp	Water	30 mL/g, 250 W	1.64	[23]
	Ethanol (60%)		1.57	
Turkey berry (*Solanum torvum* Sw)	Ethanol (≈57%)	80 °C, 49.7 mL/g, 17.3 min	192	[36]
*Opuntia ficus-indica* [L.] Mill. flowers	Ethanol (36%)	53 °C, 60 min	24.4	[50]
*Empetrum nigrum aerial parts*	Ethanol (62%)	53.3 mL/g, 42 °C, 21 min	32.2	[37]
*Tetrapleura tetraptera* fruit	Ethanol (60%)	26 mL/g, 20 min, 20 kHz, 150 W	7.05	[35]
*Berberis jaeschkeana* fruits	Methanol (80%) HCl 0.2 N	70 °C, 70 mL/g, 15 min, 50 kHz	77.5	[25]
**Food Waste**				
Spent coffee grounds	Ethanol (50%)	40 mL/g, 400 W	12.0	[41]
Waste *Syzygium cumini* leaves	Water	25 mL/g, 35 °C, 134 W, 50% duty cycle (1 min ON/1 min OFF), 9 min	78.4	[51]
Solid residue of *Apocynum venetum* tea	Methanol–Acetone	NaOH 4.29 mol/L, 15 mL/g, 60 °C, 63 min	0.3	[52]

**Table 3:** Recent applications of PLE to polyphenol extraction.

Matrix	Solvent	Extraction Conditions	TPC (mg GAE/g Dry Mass)	Reference
**Plants**				
Clove (*Syzygium aromaticum*) leaves	Ethanol	60 °C, 60 bar	20.3	[60]
Stevia leaf	Ethanol (70%)	15 mL/g, 125 °C, 30 min	310	[61]
Feijoa leaf	Ethanol (15%)	55 °C, 300 bar, 210 min	132	[62]
**Food**				
*Opuntia stricta* var. *Dillenii* wild prickly pears	Ethanol (50%)	25 °C	-	[58]
Jambolan fruit (*Syzygium cumini* L.)	Acidified ethanol (≈80%)	5000 W/L, 7.5 min	60.5	[63]
**Food Waste**				
Cranberry pomace	Ethanol (30%)	140 °C, 50 bar	85.9	[64]
Wine-making grape pomace	Ethanol (55%)	130 °C, 22 min	2.8	[65]
Açai (*Euterpe oleracea*) by-product	Ethanol (75%)	115 °C	12.0	[66]
Olive pomace	Ethanol (52%)	136.5 °C, 103 bar, 20 min	1.7	[59]

**Table 4:** Recent applications of SFE to polyphenol extraction.

Natural Resource	Solvent	Extraction Conditions	TPC (mg GAE/g Dry Mass)	Reference
**Plants**				
*Leptocarpha rivularis* stems	CO_2_-ethanol (5%)	60 °C, 500 bar	6.3	[70]
*Asparagus officinalis* L.	CO_2_-water ethanol (1:1)	65 °C, 150 bar	3.42	[77]
*Hibiscus sabdariffa*	CO_2_-ethanol (≈17%)	250 bar, 50 °C	113	[78]
Black Rosehip	CO_2_-ethanol (25%)	280 bar, 60 °C	76.6	[73]
Black poplar (*Populus nigra* L.) buds	CO_2_	60 °C, 300 bar	31.1	[74]
Rice husk	CO_2_-25% ethanol–water (50% *v*/*v*)		1.3	[75]
**Food Waste**				
Leaves of *Labisia pumila*	CO_2_-ethanol (50%)	60 °C, 20 MPa	-	[71]
Mandarin (*Clementina orogrande*) peel	CO_2_-ethanol (5–10%)	40–50 °C	19.5	[72]
Custard apple (*Annona squamosal* L.) peel	CO_2_-ethanol (12%)	52 °C, 261 bar, 54 min	109.4	[76]
Cacao pod husk	CO_2_-ethanol (≈14%)	60 °C, 299 bar	12.9	[79]
*Castanea sativa* shells	CO_2_-ethanol (15%)	60 °C, 350 bar	-	[80]
Orange pomace	CO_2_-ethanol (6%)	40 °C, 350 bar	21.8	[81]

**Table 5:** Recent applications of HVED to polyphenol extraction.

Matrix	Solvent	Extraction Conditions	TPC (mg GAE/g Dry Mass)	Reference
**Plants**				
Sage extracts (*Salvia officinalis* L.)	Ethanol (50%)	3.9 min, voltage for argon (15, 20 kV) and nitrogen (20, 25 kV)	57.21	[88]
Oregano (*Origanum vulgare* L.)	Ethanol	50% ethanol, 50 mL/g, 100 Hz, pulse duration of 400 ns, 30 mA, electrode distance 15 mm, 15 and 20 kV for argon and 20 and 25 kV for nitrogen (3 and 9 min, respectively)	191.3	[89]
**Plants**				
*Wild thyme* (*Thymus serpyllum* L.)	Ethanol (50%)	50 mL/g, 20 kV, 0.75 L min^−1^ of argon and nitrogen, 9 min	42.9	[90]
*Lycium ruthenicum*	Ethanol (20%)	pH 7, 8 min, 27 mL/min, 8 kV	-	[91]
**Food**				
*Opuntia stricta* var. *Dillenii* wild prickly pears	Ethanol (65%)	40 mL/g, 49.37 J/mL	669 *	[58]
Jambolan fruit (*Syzygium cumini* L.)	Ethanol	35 mL/g, 12 mL/min, electrode gap distance 3.1 mm, 29 kV/cm	197	[63]
**Food Waste**				
Grape pomace	Ethanol (30%)	5 mL/g, 60 °C, 30 min, 80 kJ/kg, electrodes distance of 5 mm	28	[92]
Pomegranate peels	Hot water	40 kV, distance of electrodes 40 mm, 7 min	46.0	[93]
Olive leaves	Ethanol (50%)	Argon, 9 min, 20 kV	-	[94]
Orange pomace	Water	10 min, 18 kV.	618 *	[95]

* mg/L.

**Table 6:** Recent applications of EAE to polyphenol extraction.

Matrix	Enzyme	Extraction Conditions	TPC (mg GAE/g Dry Mass)	Reference	
**Plants**					
Yerba mate (*Ilex paraguariensis A. St.-Hil*)	Carbohydrases	50 °C, enzyme concentration of 168 FGB/100 g, pH of 4.5, 120 min	3.4 *	[101]	
**Food**					
Black goji berry (*Lycium ruthenicum*)	Pectinase	1.5% (*w*/*v*) pectinase, 15 mL/g, 50 °C, 30 min	-	[91]	
**Waste**					
Eggplant peels	Cellulase	5% cellulase, 32 °C, 60 min	2040 **	[98]	
Sweet cherry (*Prunus avium* L.) pomace	Depol (36 U/mL from *Humicola* sp.)	70 °C, pH 10.0, 40 min, 90 µL of pectinase/g of sample	1.1	[99]	
	Promod (220 U/mL from *Bacillus licheniformis*)	70 °C, pH 10.0, 40 min, 90 µL of pectinase/g of sample	2.8		
	Pectinase (1060 U/mL from *Aspergillus* sp.)	70 °C, pH 10.0, 40 min, 90 µL of pectinase/g of sample	1.1		
Blackcurrant (*Ribes nigrum* L.) press cake	Cellulase	4 h	13.5	[102]	
Banana peel	Viscozyme^®^ (cellulolytic enzyme mixture; Merck KGaA, Darmstadt, Germany)	1.0% enzyme, 9 h, 55 °C, solute/liquid ratio 1:25.	25.4	[103]	
Citrus peel	Celluzyme MX (cellulase)	50 °C, enzyme 1.5% (*w*/*w*), 10 min	1.6	[104]	
Winemaking by-products	Pronase (Protease from *Streptomyces griseus*)	70% (*v*/*v*) acetone (2.5%, *w*/*v*), gyratory water bath shaker at 30 °C for 20 min	13.9	[105]	
	Viscozyme^®^ (cellulolytic enzyme mixture)		24.7		

* mg GAE/g fresh mass; ** mg GAE/L

**Table 7:** Main characteristics of extraction techniques.

Extraction Technique	General Principle	Advantages	Disadvantages	Efficiency Maximization Recommendations	References
MAE	Application of electromagnetic radiation at wavelengths in the microwave range	Extraction time reduction. Efficiency. Compatibility with natural solvents. Lower energy consumption. Scalability (safety, cost, energy control and automation).	Incompatible with non-polar solvents	Reduce pH. High temperatures (80–90 °C). Moderate power (500 W). Short extraction times (less than 3 min).	[7,10,11,12,14,108]
UAE	Application of sound waves with a frequency above the range audible to humans	Improvement on the polyphenol’s stability at optimized conditions. Cost. Efficiency.	Degradation of compounds	Moderate power Moderate temperatures (40–45 °C). Short extraction times (less than 30 min). Moderate power (less than 500 W).	[2,25,30,38,40,41]
PLE	Combining high pressures with moderate-high temperatures above the normal boiling point of solvents	Extraction time reduction. No alteration of the composition of the extracts. Easy automation and good reproducibility. Solvent amount reduction.	High cost. Coextraction of interfering substances.	High temperatures. High pressure.	[53,55,56,58,59,109]
SFE	Application of pulsed fast discharge voltages across an electrode gap below the surface of suspensions	Versatility. Extraction time reduction. Eliminating or reducing organic solvents.	Use of organic solvents as co-solvents.	Moderate temperatures (40–45 °C). Pressures of around 250 bar. Organic co-solvent content of less than 20%.	[67,71,72,73,74,75,76]
HVED	Application of pulsed fast discharge voltages across an electrode gap below the surface of suspensions	Extraction time reduction. Lower energy consumption. Fewer impurities in the extract.	Free radical production. Less selective.	Voltages of 20–25 Kv. Short extraction times (less than 10 min).	[19,82,86,87]
EAE	Treatment with an enzyme or different combinations of polysaccharide-degrading enzymes	Reducing solvent needs. Improved quality of extracted compounds. Gentler conditions. Less waste.	Alterations on the phenolic profile. Negative interference of improper enzyme activities from the plant matrix.	Enzyme loading. Longer extraction time (hours). Moderate temperatures. Select enzymes with complementary activities. Low pH.	[85,96,98,99]

**Table 8:** Comparative studies of different techniques for polyphenol extraction.

Natural Resource	Technique	Solvent	Extraction Time (min)	Temperature (°C)	Liquid-to-Solid Ratio (mL/g)	Other Parameters	Recovery Yield (%)	Reference
*Camellia japonica* var Eugenia	MAE		5	180 °C			80	[112]
	UAE	39% acidified ethanol	8	-	-	2% amplitude	56	
Sage (*Salvia fruticosa* L.) post-distillation residues	MAE	72% ethanol	15	40	30	-	24.5	[111]
	UAE	68%	10	47	10	20 kHz	23.7	
*Lantana camara* Linn. leaves	MAE	53% ethanol	15	80	50	300 W	38.6	[110]
	UAE	35% ethanol	25	30	60	37 kHz 150 W	32.5	
Blue pea flower	MAE	Water	30	50	15	800 W	37.2	[21]
	UAE					60 Hz 230 W	40.3	
	EAE					3% (*w*/*v*) Pectinase	27.8	
Black goji berry	MAE	Water	60	50	15	800 W	62.1	[22]
	UAE		30			60 Hz 230 W	45.3	
	EAE					3% (*w*/*v*) pectinase	73.6	
Clove (*Syzygium aromaticum*) leaves	PLE	Ethanol	20	60	-	-	6.6	[60]
	SFE	CO_2_	30		-	300 bar	1.9	
Feijoa leaf	PLE	Ethanol	50	80	-		35.1	[62]
	SFE	CO_2_-15% ethanol	210	55		30 MPa	32.9	
Apple seeds	UAE	Hexane	30	30	-	56 W 64% amplitude	17.2	[113]
	SFE	CO_2_	140	40	-	26 MPa	19.3	
*Dendrobium chrysotoxum* flowers	UAE		30	40	40	400 W	0.6	[114]
	SFE		90	50		20 MPa	2.0	

## Data Availability

Not applicable.
